# Composition and predictive functional analysis of bacterial communities inhabiting Chinese Cordyceps insight into conserved core microbiome

**DOI:** 10.1186/s12866-019-1472-0

**Published:** 2019-05-23

**Authors:** Fei Xia, Xin Zhou, Yan Liu, Yuling Li, Xiaohui Bai, Xuanwei Zhou

**Affiliations:** 1 Engineering Research Center of Cell & Therapeutic Antibody, Ministry of Education, and State Key Laboratory of Microbial Metabolism, and School of Agriculture and Biology, Shanghai Jiao Tong University, Shanghai, 200240 People’s Republic of China; 2 School of Food and Biological Engineering, ShaanXi University of Science & Technology, Xi’ An, 710061 China; 3 Jackson Laboratory for Genomic Medicine, Farmington, CT 06032 USA; 4 Grassland Research Institute, Qinghai Academy of Animal Sciences and Veterinary Medicine, Xining, Qinghai province 810016 People’s Republic of China

**Keywords:** Chinese Cordyceps, Bacterial communities, Composition, High throughput sequencing, Predictive functional profiling

## Abstract

**Background:**

Over the past few decades, most attention to Chinese Cordyceps-associated endogenous microorganism was focused on the fungal community that creates critical bioactive components. Bacterial community associated with Chinese Cordyceps has been previously described; however, most studies were only presenting direct comparisons in the Chinese Cordyceps and its microenvironments. In the current study, our objectives were to reveal the bacterial community structure composition and predict their function.

**Results:**

We collected samples of Chinese Cordyceps from five sites located in the Qinghai-Tibet Plateau and used a high throughput sequencing method to compare Chinese Cordyceps-associated bacterial community composition and diversity quantitatively across sites. The results indicated that for the Chinese Cordyceps-associated bacterial community there is no single core microbiome, which was dominated by the both Proteobacteria and Actinobacteria. Predictive functional profiling suggested a location specific function pattern for Chinese Cordyceps and bacteria in the external mycelial cortices involved in the biosynthesis of active constituents.

**Conclusions:**

This study is firstly used high throughput sequencing method to compare the bacterial communities inhabiting Chinese Cordyceps and its microhabitat and to reveal composition functional capabilities of the bacteria, which will accelerate the study of the functions of bacterial communities in the micro-ecological system of Chinese Cordyceps.

**Electronic supplementary material:**

The online version of this article (10.1186/s12866-019-1472-0) contains supplementary material, which is available to authorized users.

## Background

Chinese Cordyceps, a parasitic complex of fungus and caterpillar, has been used for medicinal purposes with a long history in China and other Asia East countries [1]. Because of their special growth pattern and distribution area, little is known for their growth process and the mechanism of active component metabolism [2, 3]. Although the family of Cordyceps includes over 500 species that are pathogens of arthropods, Chinese Cordyceps was only found to grow in the Qinghai-Tibetan Plateau and its surrounding regions with 3000~5000 m altitude, including Qinghai, Tibet, Yunnan, Gansu and Sichuan provinces in China and in certain areas of the southern flank of the Himalayas, such as Nepal, India and Bhutan, etc. [4]. Approximately 96.5% of the Chinese Cordyceps habitats distributed in China, production yields harvesting from Qinghai and Tibet were approximately accounting for 71.4% of the world’s total harvesting [5]. Therefore, Qinghai and Tibet should be the main producing areas of Chinese Cordyceps. In general, Chinese Cordyceps quality is relating to varieties of factors, such as geographical conditions, climatic conditions, and cohabitation microorganisms. Among these factors, the inhabiting microorganisms are supposed to be one of the main factors that could influence on the quality of Chinese Cordyceps [6]. Many scientists have made great efforts in artificially cultivating Chinese Cordyceps in recent years. Even the artificial cultivation of Chinese Cordyceps achieved some success [7], however, there’s still a lot of confusion in the mechanism of the Chinese Cordyceps formation process till now. The possible reason may be the function and the population of a lot of other cohabitation microorganisms inhabiting in Chinese Cordyceps, including its parasitic bacteria, companion fungus or symbiotic bacteria [8], was not clear beside the fungus *Ophiocordyceps sinensis*.

In recent years, a lot of work revolved around the microorganism communities living in Chinese Cordyceps was conducted. The fungal community associated with Chinese Cordyceps had been described by a number of cultures-dependent and independent methods [9–15]. In spite of we reported that there were multiple fungi and bacteria inhibiting Chinese Cordyceps and its microhabitat [10]. The publication involved in the analysis of the diversity of bacteria in the native habitat of Chinese Cordyceps was very little [16]. However, bacteria inhabiting this small ecosystem may play a role of “pioneer species” when fungus *O. sinensis* infecting the larva. In the previous studies, we tried to set up an effective method for the molecular analysis of the bacterial microbiota inhibiting Chinese Cordyceps [10, 11], and other research also involved in investigating the intestines bacterial community of *Hepialus gonggaensis* larva [17] and soil bacterial communities in Chinese Cordyceps habitat [16]. However, those investigations only were the description of the bacterial community composition, and the function of those bacteria was still not being referred.

**Fig. 1:**
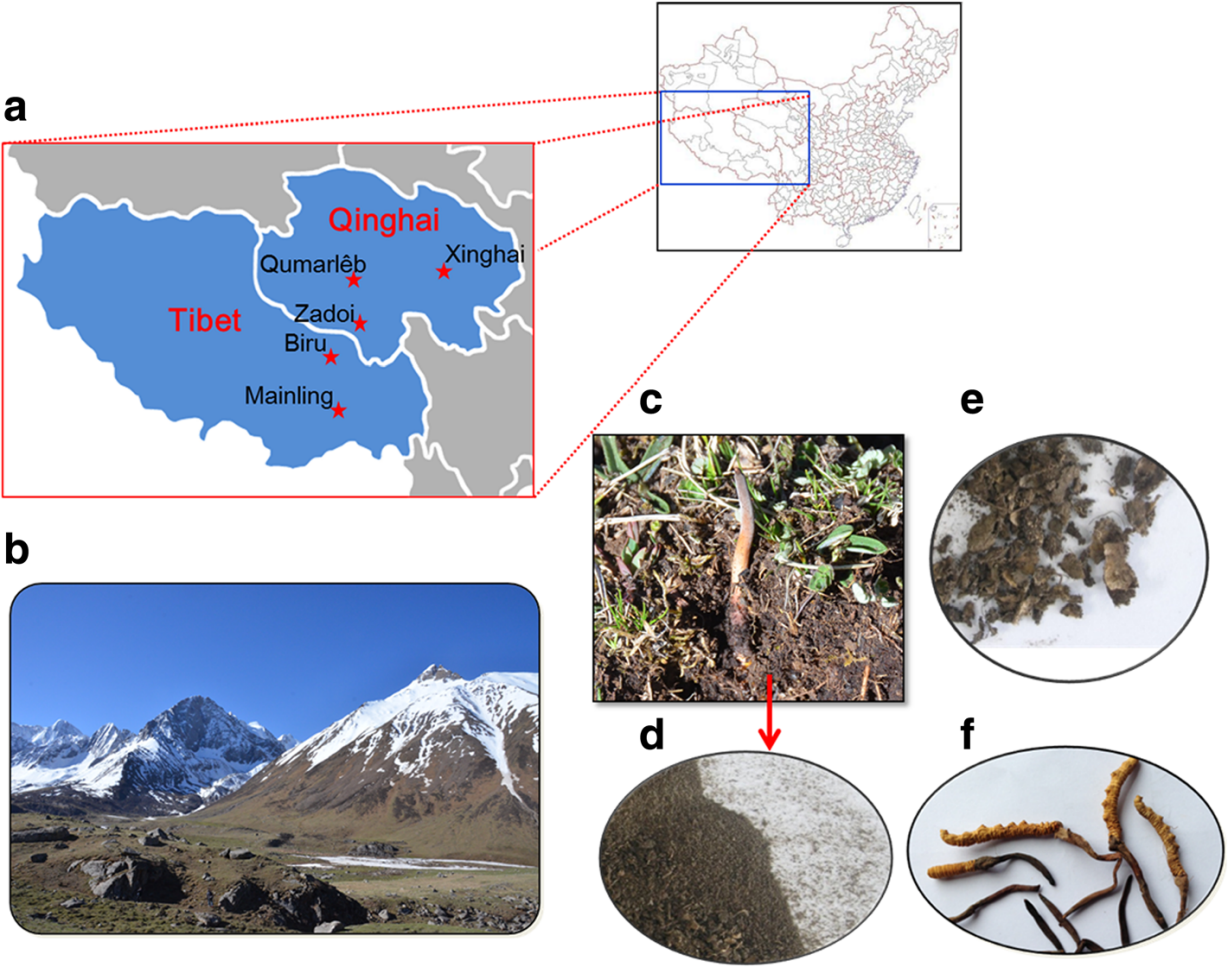
Chinese Cordyceps samples collection and pretreatment. **a** Locations of the sampling areas including Counties of Qumarlêb, Xinghai County and Zadoi of Qinghai province were abbreviated as “Q”, “X” and “Z”, respectively. Biru county of Nagqu and Mainling County of Nyingchi City of Tibet Autonomous Region were abbreviated as “Na” and “Ny”, respectively (the map was depicted by Fei Xia); Environment and habitats of Chinese Cordyceps growth (**b** and **c**); collected Chinese Cordyceps samples were divided into microhabitat soil (soil adhering to the surface of the membrane covering Chinese Cordyceps, short for “S”) (**d**), mycoderm (microhabitat including external mycelial cortices that cover larvae, short for “M”) (**e**) and fruiting body (fruiting body including stromata and sclerotia, short for “F”) (**f**). Zhou XW took the photograph with a regular digital camera

We hypothesize that Chinese Cordyceps and its microhabitat soil could be regarded as a microecosystem which is composed by fungi and bacteria. Bacterial community may play a key role in formation of the Chinese Cordycep, including the process that fungi *O. sinensis* infect the larva and the secretion of active compounds by the Chinese Cordyceps. Our objective was to reveal the bacterial community in Chinese Cordyceps and its microhabitat soil with a high-throughput sequencing method, while to analyze the predictive functional profiling [18] which had been widely used to study bacterial communities from various environments [19–21]. To do this, Chinese Cordyceps and soil samples were collected from five different geographic areas of its core production area: Qinghai province and Tibet Autonomous Region (Fig. 1). The current research was among the few studies focused on the bacterial community in Chinese Cordyceps and its microhabitat soil. In addition, bacterial community analysis and the function prediction would help us to reveal the formation of Chinese Cordyceps and its active compounds secretion. Concerning the function prediction of bacterial community could help us to enhance the artificial cultivation of Chinese cordyceps.

## Results

### Alpha- diversity of bacterial community cohabitated to Chinese Cordyceps

Totally, 591,672 high quality reads were obtained in the current study with the minimum 24,204 reads in sample XS. The Good’s coverage index of the sample (Table 1) and the rarefaction curves (Additional file 1: Figure S1) indicated perfect evidence to the conclusions. Sampling depth of sequence data set in the current study was enough, because the operational taxonomic units (OTUs) number did not increase with the growth of sequencing depth (Additional file 1: Figure S1A, B, and C) and the Shannon index (indicating the diversity of the bacterial community) flatten out after reads number more than about 2,000 in each of samples (Additional file 1: Figure S1D, E, and F).

**Table 1:** Indices of bacterial community in samples of Chinese Cordyceps

	Shannon^c^			Simpson			Chao			ACE			Coverage		
	F^b^	M	S	F	M	S	F	M	S	F	M	S	F	M	S
Q^a^	3.23	3.12	3.26	0.07	0.09	0.08	107.00	102.50	107.25	106.06	104.38	102.89	0.9996	0.9997	0.9988
X	2.78	3.31	3.72	0.15	0.07	0.04	154.00	100.27	143.13	149.52	102.55	141.42	0.9989	0.9987	0.9989
Z	3.43	2.92	3.24	0.07	0.14	0.06	145.50	109.00	139.15	143.91	102.17	139.89	0.9996	0.9992	0.9986
Na	3.49	3.57	2.55	0.06	0.04	0.18	135.09	115.50	125.25	135.27	111.35	125.17	0.9987	0.9982	0.9988
Ny	3.71	2.96	3.13	0.04	0.11	0.07	173.00	117.30	79.80	133.00	119.73	84.81	0.9985	0.9982	0.9992

^a^Abbreviation of the sampling sites. Counties of Qumarlêb, Xinghai County and Zadoi of Qinghai province were abbreviated as “X”, “Q” and “Z”, respectely. Biru county of Nagqu and Mainling County of Nyingchi City of Tibet Autonomous Region were abbreviated as “Na” and “Ny”, respectively

^b^Abbreviation of different samples of natural Chinese Cordyceps. Fruiting body of Chinese Cordyceps, external mycelial cortices and soil adhere to Chinese Cordyceps were abbreviated as “F”, “M” and “S”, respectely

^c^All the indexes were calculated after the reads were normalized to minimum (24204 reads) in each sample

The alpha diversity of the bacterial community in each Chinese Cordyceps sample was revealed by indices of Shannon, Simpsons, Chao1, and ACE (Table 1). Before these indices were calculated, reads number in each sample was normalized to the minimum (24,204 reads). We compared the difference between some samples of Chinese Cordyceps collected from the five areas with unpaired two-tailed Student’s t-test. Diversity indices of bacterial communities, including Shannon and Simpsons, did not show a significant difference (*p* > 0.05) among the sample groups of fruiting body (F), external mycelial cortices (M) and soil (S). On the contrary, abundant indices of bacterial community (including Chao and ACE) indicated a significant difference among the sample groups of Chinese Cordyceps. Chao index of the bacterial community in sample F group was significantly higher than in M samples group collected from five areas (*p* < 0.05), however, there was no significant difference between sample F group and S group (*p* = 0.17). In addition, ACE index of sample F group was significant higher than samples M group (*p* < 0.05), however, there was no significant difference of the ACE index between sample F groups and sample S groups (*p* = 0.30). In conclusion, the abundance of bacterial community in fruiting body (F) of Chinese Cordyceps was much higher than in external mycelial cortices (M) and soil (S) samples.

### Bacterial community composition

**Fig. 2:**
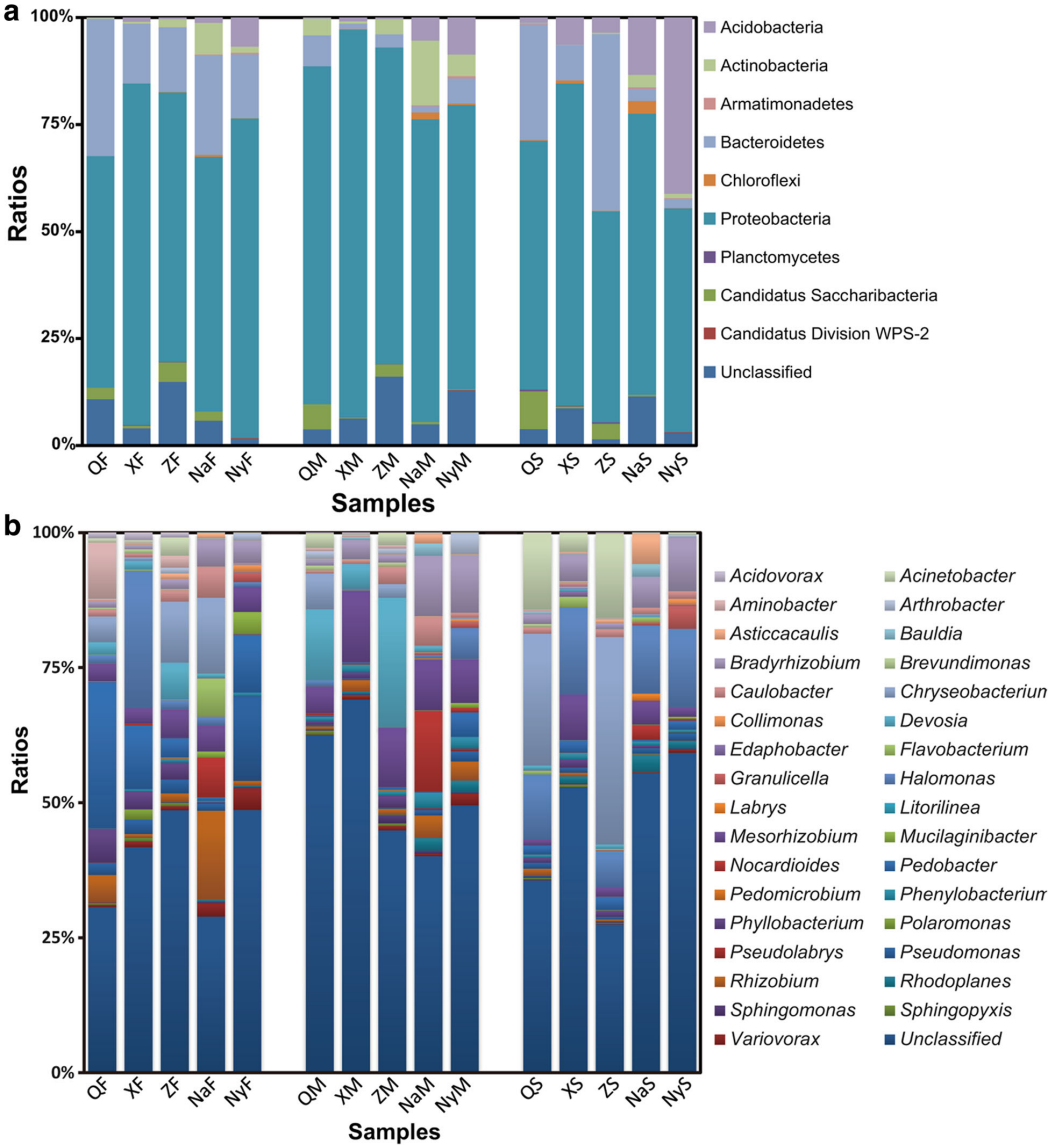
Bacterial community structures at phylum (**a**) and genus (**b**) level inhabiting in Chinese Cordyceps and its microhabitat soil samples. Samples name were the same as described in Fig. 1

Bacterial community’s composition of Chinese Cordyceps samples at the phylum and genus level was analyzed respectively. Based on the classifiable sequences, 9 phyla were identified across the entire sample. The dominant phyla were Planctomycetes, Proteobacteria, Chloroflexi, Bacteroidetes, Armatimonadetes, Actinobacteria and Acidobacteria and two candidate phyla. Proteobacteria was the most abundant phylum in all Chinese Cordyceps samples (Fig. 2a). Its proportion was ranged from 49.28% (in ZS) to 90.50% (in XM) in the collected samples. The proportion of Proteobacteria in external mycelial cortices (M) samples was significantly higher than in microhabitat soil (S) samples (*p* < 0.05). Bacteroidetes was another abundant phylum in samples of the fruiting body (F) and microhabitat soil (S) with its average proportion was 19.86 and 16.30%, respectively. However, the proportion of Bacteroidetes in external mycelial cortices (M) samples was significantly lower than in the fruiting body (F) samples (*p* < 0.05). In addition, Actinobacteria was also a dominant bacteria in fruiting body samples (F) and samples of external mycelial cortices (M). It was worth mentioning that proportion of Acidobacteria in Nyingchi City of Tibet Autonomous Region (Ny) was several times higher than in the samples collected from other areas.

More than 60 genera were found in all Chinese Cordyceps samples, which distributed differently in all the samples (Fig. 2b). Similar to other bacterial community researches, “Unclassified microbe” made up a sizeable proportion. In fruiting body samples (F), average proportion of *Pedobacter* was 10.78%. Its proportion reached 27.10% in the samples of QF, which was several time higher than in fruiting body collected from other areas. The proportion of *Halomonas* in the sample of XF reached 25.44%, which was also higher than another four areas’ sample. In addition, average proportions suggested that *Chryseobacterium* (6.17%), *Rhizobium* (4.95%), *Pseudomonas* (4.89%) and *Mesorhizobium* (4.18%) were the relatively abundant of bacteria in the fruiting body samples. For samples of Chinese Cordyceps external mycelial cortices (M), average proportions indicated that *Mesorhizobium* (9.29%) and *Devosia* (8.51%) were rather abundant bacteria. For instance, proportion of *Mesorhizobium* ranged from 4.88% (in QM) to 13.16% (in XM). The proportion of *Devosia* was 23.91% in sample ZM and 13.05% in sample QM. Unpaired two-tailed Student’s t-test indicated that proportion of *Mesorhizobium* in Chinese Cordyceps external mycelial cortices samples was significantly higher than in fruiting body and microhabitat soil samples (*p* < 0.05). In addition, genus of *Bradyrhizobium* (5.53%), *Nocardioides* (3.25%), *Rhizobium* (2.29%) and *Caulobacter* (2.19%) were the comparatively abundant bacteria. Finally, in microhabitat soil samples (S), proportion of *Chryseobacterium* was rather higher, with 24.41% in QS and 38.40% in ZS. Average proportion value indicated that *Halomonas* (12.37%), *Acinetobacter* (6.81%), *Bradyrhizobium* (4.68%) and *Mesorhizobium* (3.50%) were also the remarkably abundant genera.

### Comparison of Chinese Cordyceps-inhabiting bacterial communities

**Fig. 3:**
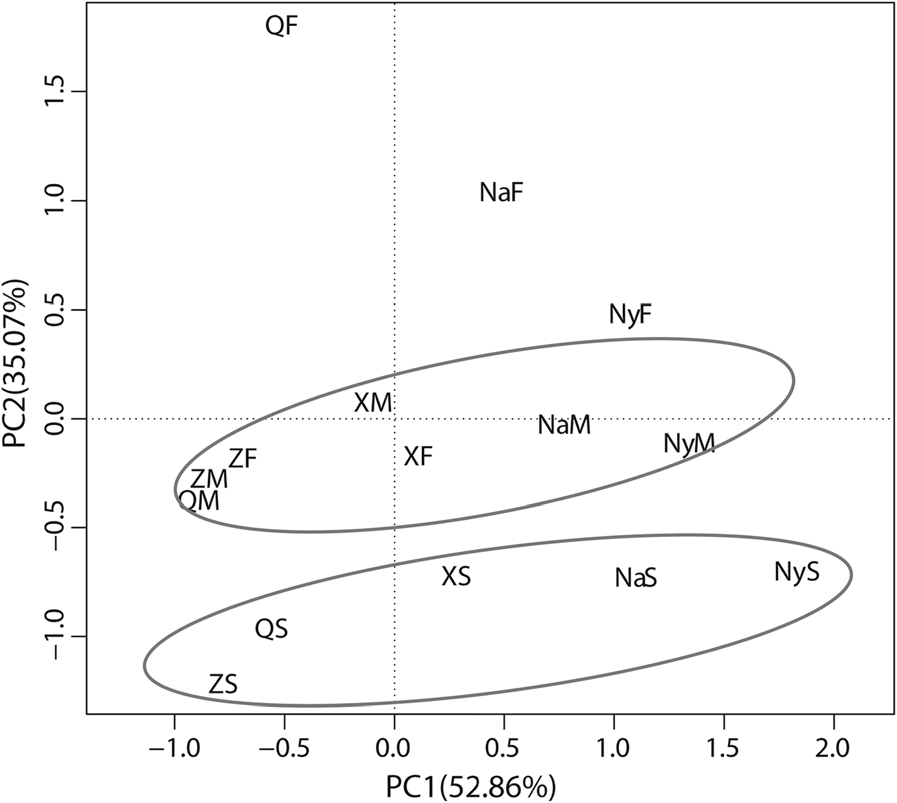
Principal component analysis of bacterial community in the samples of Chinese Cordyceps. The PCA were conducted based on the weighted normalized unifrac distance. Samples name were the same as described in Fig. 1

**Fig. 4:**
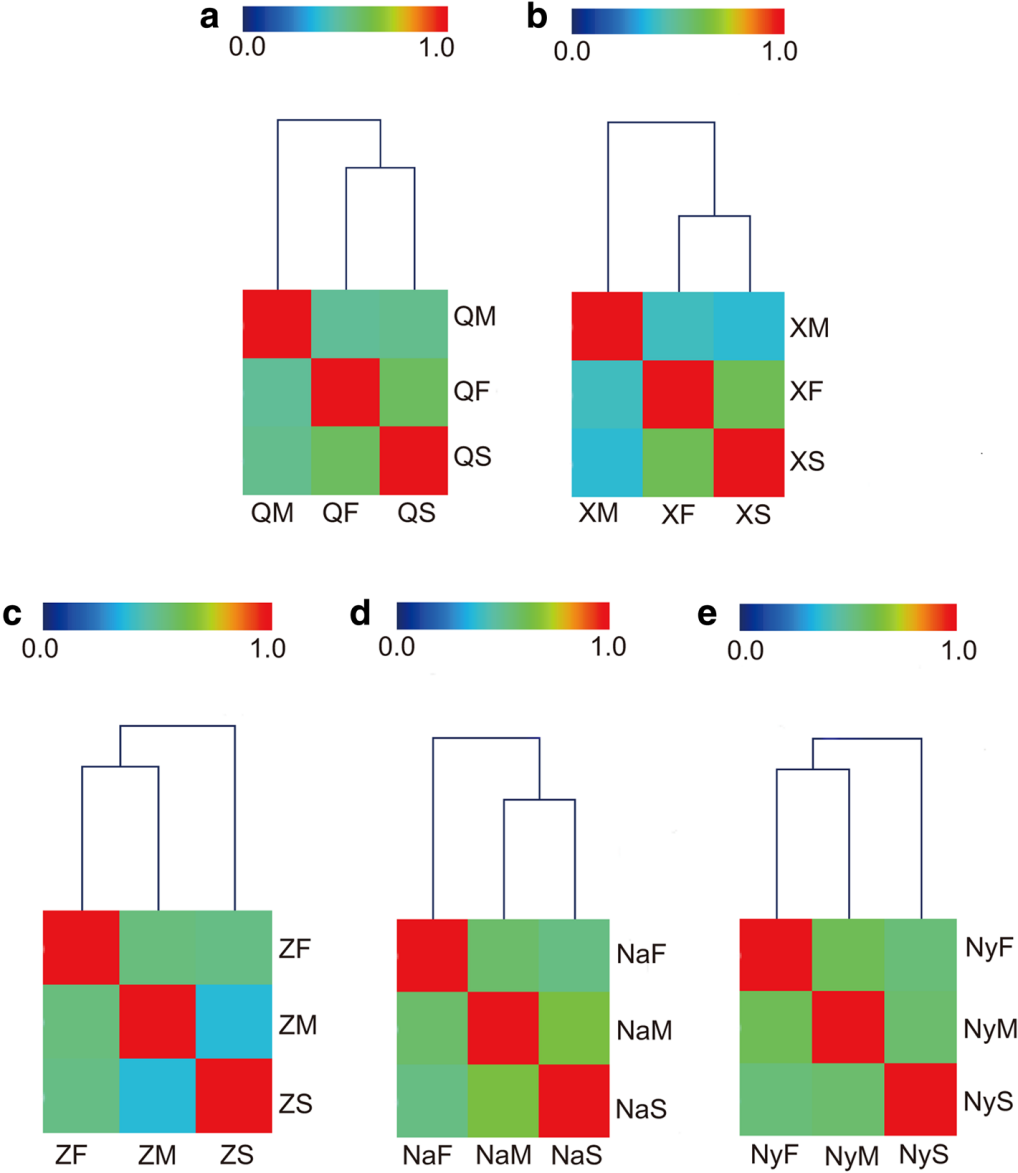
Hierarchical-clustering graphics of the weighted UniFrac distance of bacterial community inhabiting Chinese Cordyceps. The dendrograms were carried out based on the similarity matrix. **a** Chinese Cordyceps collected form County of Qumarlêb, **b** Chinese Cordyceps collected form Xinghai County, **c** Chinese Cordyceps collected form Zadoi County of Qinghai province; **d** Chinese Cordyceps collected form Biru county and Mainling **e** County of Tibet Autonomous Region

Bacterial communities in samples of Chinese Cordyceps collected from five areas were compared based on the weighted UniFrac similarity matrix. The Principal Component Analysis (PCA) indicated that soil samples collected from the five areas were separated from the samples of fruiting body and external mycelial cortices (Fig. 3), indicating composition of bacterial community in soil sample was different from that in fruiting body and external mycelial cortices samples. This phenomenon was similar with the fungal community published before [9]. Bacterial community in samples of the fruiting body, external mycelial cortices, and microhabitat soil showed various patterns in five sampling areas (Fig. 4). The bacterial community similarity among each sample of Chinese Cordyceps collected from five areas was shown in Additional file 4: Table S1. Bacterial community in microhabitat soil and fruiting body collected from Counties of Qumarlêb was clustered together with the similarity value of 0.5863 (Additional file 4: Table S1) and the sample of QM formed an out-group (Fig. 4a). This pattern was the same ones as samples of Chinese Cordyceps collected from Xinghai County (X) (Fig. 4b). However, the bacterial community in samples of Chinese Cordyceps collected from Zadoi (Z) of Qinghai province and Mainling County of Nyingchi City (Ny) of Tibet Autonomous Region suggested a different pattern. Bacterial communities were more similar in the fruiting body and external mycelial cortices so that these two samples were grouped together and the microhabitat soil formed an out-group (Fig. 4c and e). Finally, in the Chinese Cordyceps collected from Biru county of Nagqu (Na) of the Tibet Autonomous Region, samples of external mycelial cortices and microhabitat soil clustered together and the sample of the fruiting body were laid at outside of the cluster tree (Fig. 4d).

### Abundance of Chinese Cordyceps-inhabiting bacterial communities

**Fig. 5:**
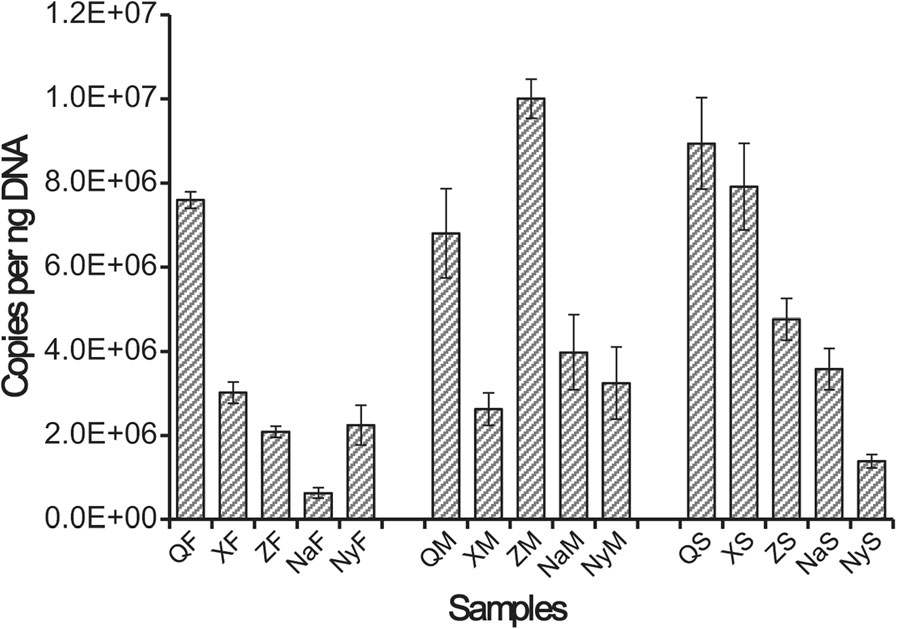
Abundance of Chinese Cordyceps-inhabiting bacterial communities. Samples name were the same with described in Fig. 1. The error bars indicate SDs (*n* = 3)

The abundance of bacterial communities of all the samples was represented by the copy number of 16S rRNA gene sequences per nano-gram (ng) of genomic DNA. Totally, the average copy number of 16S rRNA gene in all Chinese Cordyceps samples was decreased as the latitude increased with the exception of ZM (Fig. 5). The average copy number of 16S rRNA gene in Chinese Cordyceps fruiting body samples was ranging from 6.31 ± 0.93 × 10^5^ (in NaF) to 7.60 ± 0.19 × 10^6^ (in QF) copies per ng DNA. A T-test indicated that the copy number of 16S rRNA gene in QF was significantly higher and in NaF was considerably lower than in other fruiting body samples (F) (*p* < 0.05). For the samples of external mycelial cortices (M), the average copy number of 16S rRNA gene was ranging from 2.63 ± 0.39 × 10^6^ (in XM) to 1.00 ± 0.05 × 10^7^ (in ZM) copies per ng DNA. The copy number of 16S rRNA gene in XM was significantly lower than in other samples and in ZM was significantly high (*p* < 0.05). The copy number of 16S rRNA gene in microhabitat soil samples was ranged from 1.39 ± 0.16 × 10^6^ (in QS) to 8.94 ± 1.09 × 10^6^ (in NyS) copies per ng DNA. T-test results suggested that the copy number of the 16S rRNA gene was significantly lower in the sample of NyS.

### Predictive functional profile of Chinese Cordyceps-inhabiting bacterial communities

**Fig. 6:**
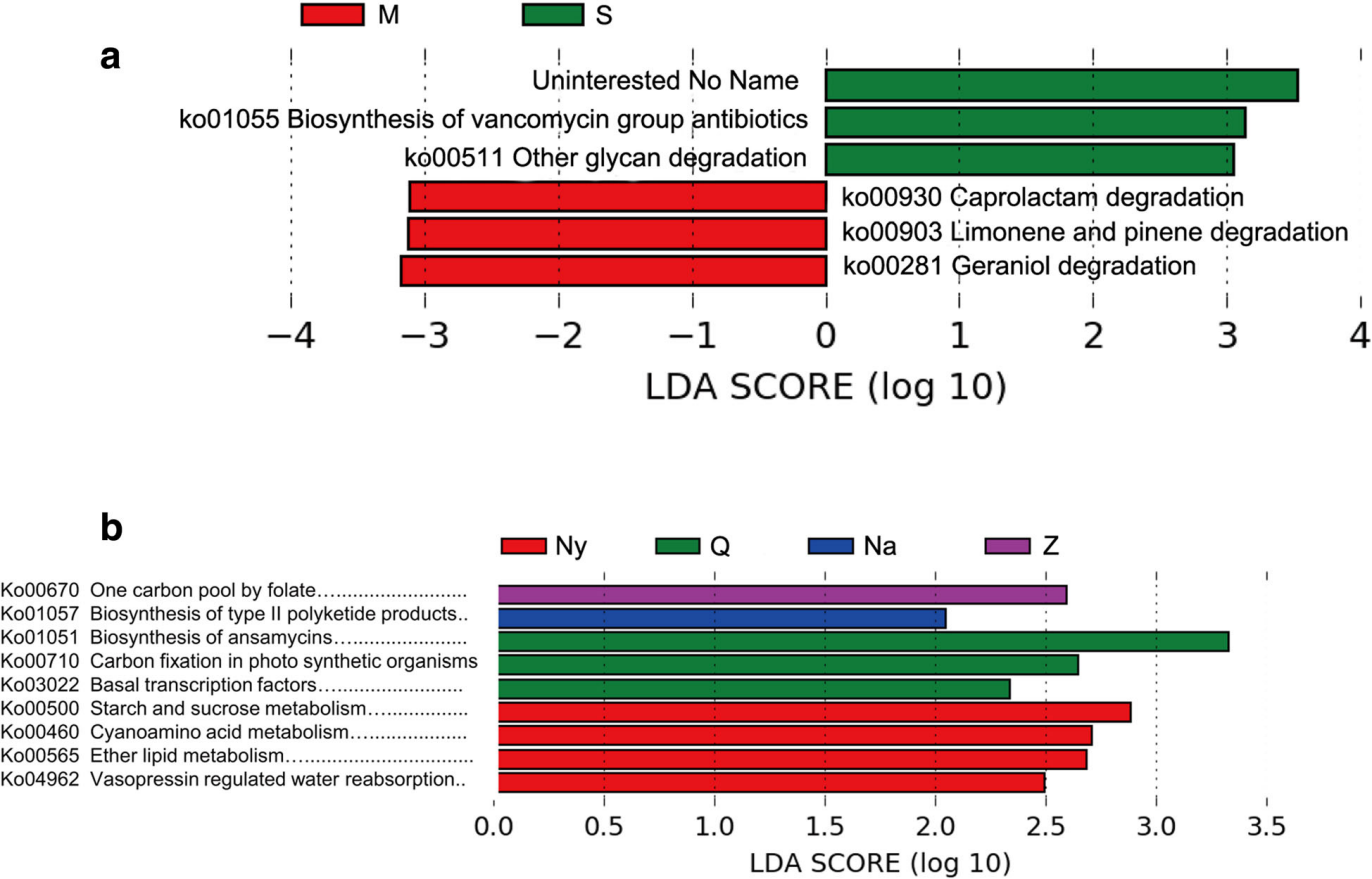
Differentially presented pathways among samples of Chinese Cordyceps (**a**) and collected areas (**b**) predicted by PICRUSt. Samples name were the same with described in 1. LDA score (log 10) higher than 2.0 to be considered significant

For pathways prediction, microhabitat soil and external mycelial cortices samples were aggregating into two separate clusters. Bacteria indicated the pathways of biosynthesis of vancomycin group antibiotics and glycan degradation were significant abundant in soil samples. In addition, caprolactam degradation, limonene and pinene degradation and geraniol degradation pathways in external mycelial cortices samples were significantly higher (Fig. 6a). There was no clear cluster based on the collection locations of those samples (Fig. 6b). However, the biosynthesis of type II polyketide products pathway in samples collected from Biru County of Nagqu Prefecture was rather abundant. The biosynthetic pathway of ansamycins was indicated rather abundant in samples collected from Qumarlêb County. In samples collected from Nyingchi, the pathway of starch, sucrose, cyan amino acid and ether lipid metabolism was also demonstrated significant distinguishing with other samples based on the LDA (liner discriminate analysis) score (Fig. 6b). This indicated that the predicted function of the bacteria population was more related to the material than the habitat location. It also comes to our attention that samples collected from Mainling County of Nyingchi City of Tibet Autonomous Region (Ny) seemed to have a different functional prediction on all material type. These results indicate habitat of Chinese Cordyceps in Mainling County of Nyingchi City of Tibet Autonomous Region was biologically significantly different from other four locations. In figures with pathway heatmap, with some exceptions, samples of the fruiting body, mycoderm and microhabitat soil were separated (Additional file 2: Figure S2). Red clusters were functions enriched in microhabitat soil samples. It involved metabolism of fatty acid, beta-alanine, tryptophan, propanoate, phenylalanine, glutathione, glyoxylate and dicarboxylate, D-arginine and D-ornithine, etc. Blue cluster were those functions enriched in external mycelial cortices (M) samples, including biosynthesis of polyketide sugar unit, lipopolysaccharide, tetracycline, phenylalanine, tyrosine and tryptophan, lysine, peptidoglycan, folate, and zeatin. The primary difference between the red and blue cluster is: there is more degradation function involved in the red cluster, and more biosynthesis function involved in the blue cluster.

**Fig. 7:**
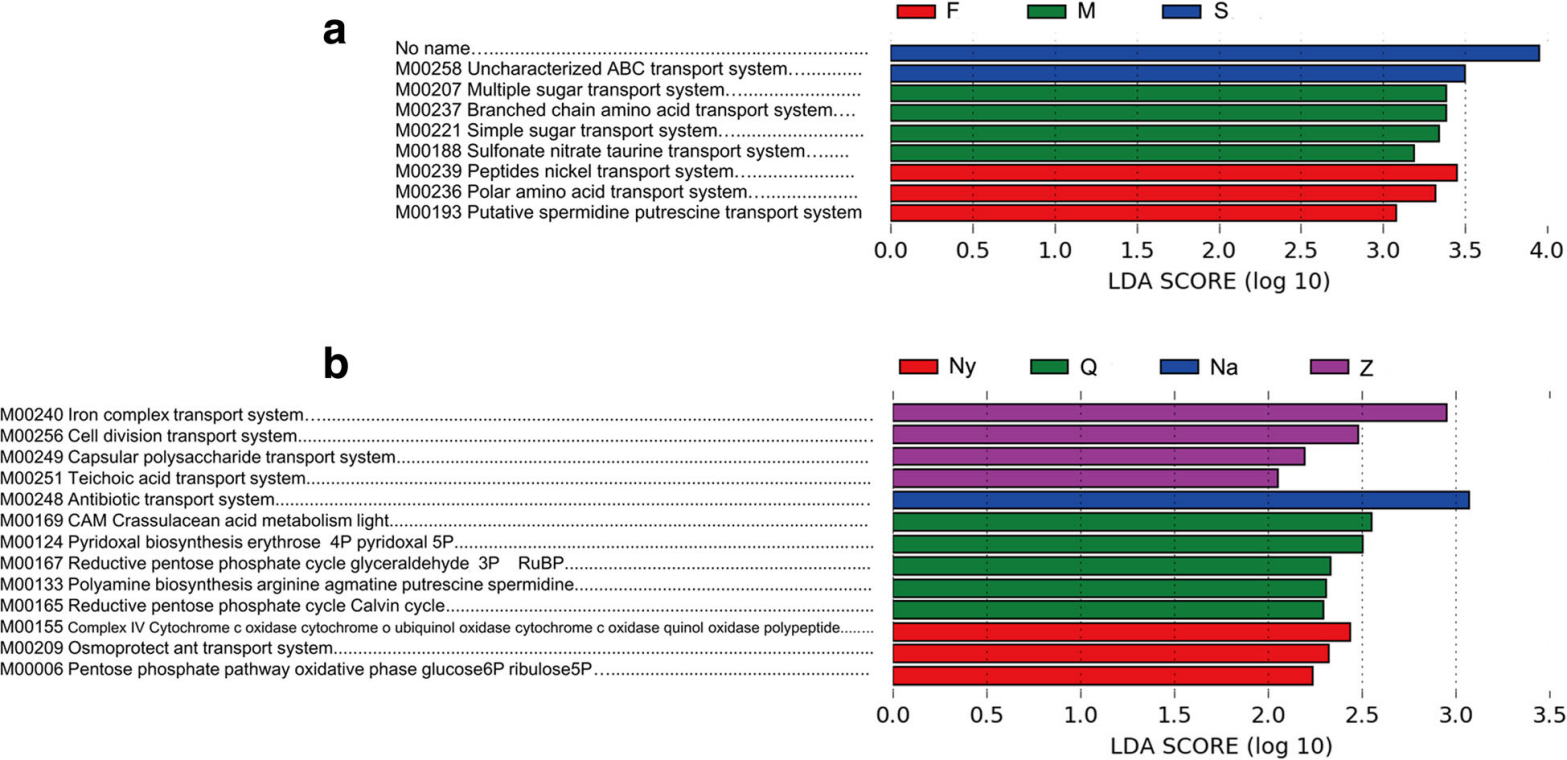
Differentially presented modules among samples of Chinese Cordyceps (**a**) and collected areas (**b**) predicted by PICRUSt. Samples mame were the same with described in 1. LDA score (log 10) higher than 2.0 to be considered significant

Modules prediction was also performed using PICRUSt (Phylogenetic Investigation of Communities by Reconstruction of Unobserved States, http://picrust.github.com/picrust/), a bioinformatics software used to predict functional metagenomes from 16S rRNA gene profiling. There were not well-separated clusters in the modules prediction of Chinese Cordyceps samples and the sampling areas profile. However, in modules prediction profile of different samples, some systems including sugar, branched chain amino acid and sulfonate nitrate taurine transport were significant abundant in external mycelial cortices (M) samples. Correspondingly, peptides nickel, polar amino acid and spermidine putrescine transport system were demonstrated more abundant in soil samples (Fig. 7a). Modules prediction profile was not formed a clear cluster among the sampling areas. There also some transport and metabolism system was demonstrated more abundant in each sampling area, respectively (Fig. 7b). In the heatmap figure of module prediction (Additional file 3: Figure S3), samples from location Counties of Qumarlêb (Q), Xinghai County (X) of Qinghai province were clustered differently with samples from Zadoi County of Qinghai province (Z), Biru county of Nagqu (Na) and Mainling County of Nyingchi City (Ny) of Tibet Autonomous Region. This indicated a location specific function pattern for Chinese Cordyceps. It also confirmed that sample from location Mainling County of Nyingchi City (Ny) of Tibet Autonomous Region is significantly different from samples collected in other location.

## Discussion

In the previous studies of microorganism community related to Chinese Cordyceps, investigator was mainly focused their interesting on fungal community, including the fungus *O. sinensis* and other fungi inhabiting in Chinese Cordyceps. One of the purposes was to produce similar substances similar to Chinese Cordyceps or discover new bioactive substances from the isolated fungi [12, 13, 22, 23]. However, there were not studies focused on Chinese Cordyceps-inhabiting bacterial community and their functions in the growth, development and formation metabolites of Chinese Cordyceps. Only a few papers were involved in Chinese Cordyceps-inhabiting bacterial community [10, 11, 17]. There are some shortcomings in these investigations, such as limitations in sampling point, technique level and analysis method, etc. Recently, meta-transcriptomics analysis of Chinese Cordyceps demonstrated that massive transcriptions of the Class I type of retrotransposons and Class II type of DNA transposons were contributing to environmental adaptation of Chinese Cordyceps, active expression of these transposable elements (TEs) could drive the rapid evolution of fungal genomes [15]. Based on the above-mentioned results, it is difficult to assess the diversity of complex bacterial communities in Chinese Cordyceps and its microhabitat soil because of the limitations of the investigation methods and sampling points. In the current study, locations of sample collection sites were mainly range from the two main production areas (Fig. 1). Subsequently, high-throughput sequencing technologies and 16S rRNA gene sequences were employed for analysis of the bacterial communities. Predictive functional profiling of microbial communities was carried out using a computational approach. Therefore, this study could more realistically reflect the situation of Chinese Cordyceps-inhabiting bacterial communities, while the bacterial population in the microhabitat soil.

Compared with a previous publication, dominate bacterial communities inhabiting intestines of *H. gonggaensis* [16] were not detected in this study. We guess that the main reason for the difference is because *H. gonggaensis* larva used for investigating the gut microbiota in the previous investigation is an artificial cultivation of larvae. But Chinese Cordyceps is a complex integrated micro-ecological system consisting of a dead body of *Hepialus* sp. and multiple intrinsic microorganisms. Using the “next-generation sequencing technology”, the results gave a comprehensive comprehension to the Chinese Cordyceps-inhabiting bacterial communities. Abundance of bacterial communities in the samples of the fruiting body (F) was much higher than in another two samples by the indices of bacterial community’s abundance, including Chao and ACE (Table 1). Stromata and sclerotia were the main section of the Chinese Cordyceps, however, there was much discrimination with stromata and sclerotia. With full of nutrients and moisture content, larva of *Hepialidae* intestines offered favorable condition for the bacterial community growing, with the formation of the Chinese Cordyceps, larva of *Hepialidae* turned into sclerotia. This would be the reason why the bacterial community abundance was higher in the samples of fruiting body.

**Fig. 8:**
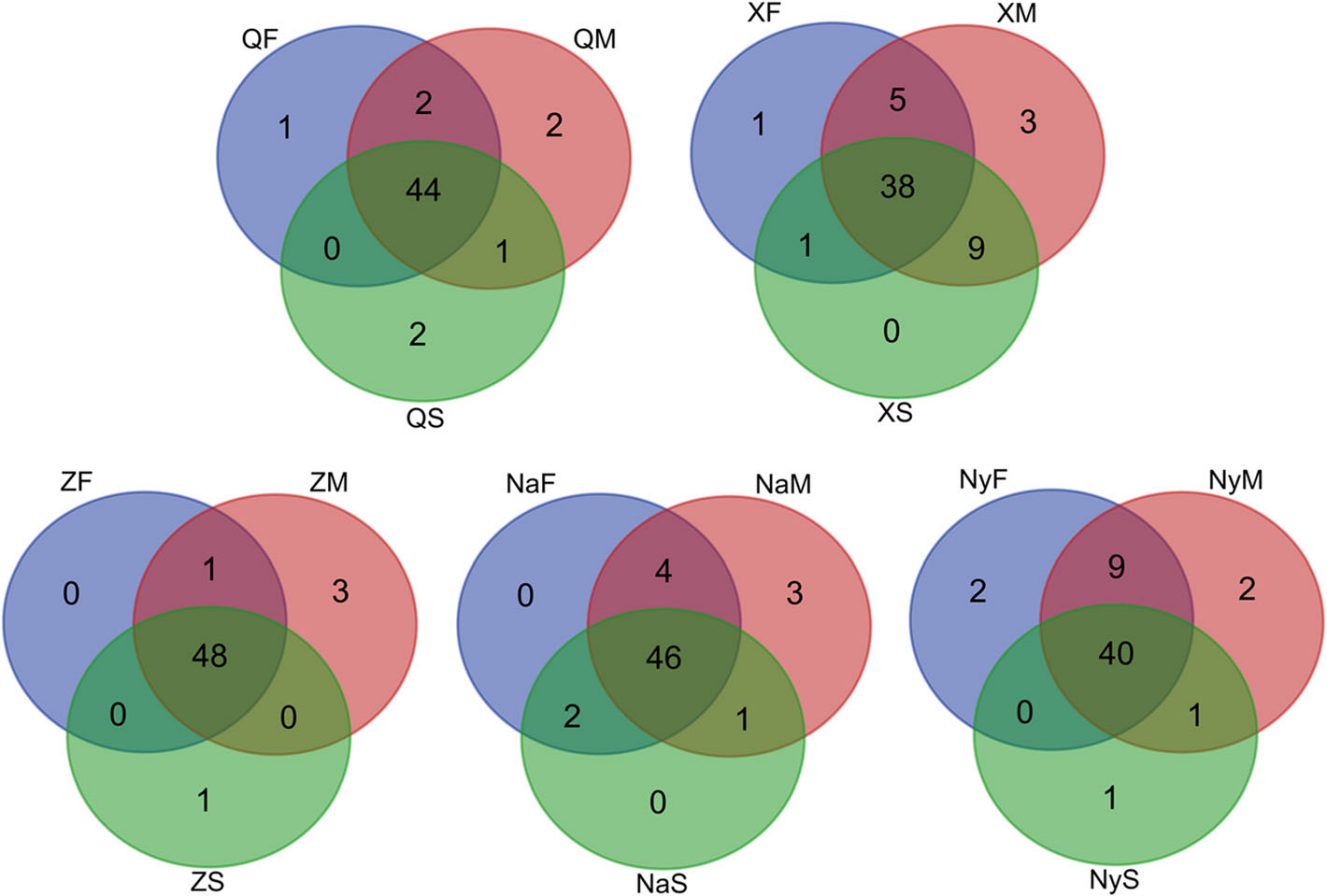
Comparison of Chinese Cordyceps-inhabiting bacterial communities collected from different locations. Samples name were the same with described in Fig. 1

Amazingly, bacterial community was rather abundant in different tissue samples of Chinese Cordyceps, which was only demonstrated by a few researchers [10, 11]. Similarity analysis of bacteria diversity of sample from different habitats was carried out in this study (Fig. 8), most of the bacterial genera were detected in different samples collected from different geographical region. Compared with the fungal communities [9], bacterial communities were more ubiquitous in different samples of the Chinese Cordyceps. It has been accepted that, microbes always play an important role in natural process, for instance, microbes in plants rhizosphere soil always change the nutrients, phytohormone, water and other biological substance, which could enhance the nutritional substance absorption [24], regulate plants’ immune system, and improve the viability of plants in extreme environments [25]. Similarly, bacterial communities inhabiting Chinese Cordyceps and its microhabitat soil may also play an important function in the formation of Chinese Cordyceps and the active components.

In the process of growth and development of Chinese Coryceps, the skin color of the *Hepialidae* larva gradually changes from white to yellow accompanied by the process of *Hepialidae* larva turn into “stiff worm”. It is entirely possible that this change is due to microbes inhabiting Chinese Cordyceps. *Chryseobacterium* bacteria, detected in the current study, are one of common pathogens of nosocomial infection, producing yellow pigment in the oxidase test [26]. Existence of *Chryseobacterium* bacteria would be the reason for the yellow skin of sclerotia of natural Chinese Cordyeps. Besides, the genus *Rhizobium and Mesorhizobium* was no doubt to related with the host larva’s food, which were the roots of some plants including a part of the genus *Polygonum*, *Astragalus, Kobrasia* and etc., such as *Kobrasia pygmaea, K. humilis, Polygonum viviparum, P. capitatum and P. macrophytum*, etc. [27]. In the current study, diversity of a bacterial community inhabiting Chinese Cordyceps was rather high. The high diversity of the bacterial community may indicate its key role in generating of Chinese Cordyceps and secreting active compounds. However, detail functions of bacterial community inhabiting Chinese Cordyceps still need to be discovered in further studies.

For predictive functional profiling of Chinese Cordyceps-inhabiting bacterial communities, samples of microhabitat soil and external mycelial cortices of Chinese Cordyceps were aggregated into two separated clusters. However, there was no clear cluster based on the collection location of those samples. This phenomenon indicated that the predicted function of the bacteria population was more related to the Chinese Cordyceps material than its habitat location. In the current study, bacterial community composition and their function prediction were rather different in Chinese Cordyceps and its microhabitat soil. Similar to our studies published before, fungal community composition in Chinese Cordyceps was much distinction in its microhabitat soil [9]. Couple with the results in the current study, we demonstrated again that the Chinese Cordyceps was a natural organic micro-ecosystem which could resist external influence of the organisms in the environments. In addition, the resistance of Chinese Cordyceps to other microorganism may also relate with the antibacterial activity of Chinese Cordyceps itself or the fungus *O. sinensis* [28]. There’s still an interesting question that needs to be studied in further, as described before, plants roots of *Polygonum*, *Astragalus, Kobrasia* were the food of the host larva, bacterial community composition and their function prediction in these plants roots and Chinese Cordyceps’ fruiting body should be focused on in the future.

In addition, results of bacterial community function prediction revealed that the function of soil microbiome in Mainling County of Nyingchi had some difference of pathways from the others (Additional file 2: Figure S2 and Additional file 3: Figure S3). Different soil bacteria function may suggest some special effecting of bacteria on the good quality of Chinese Cordyceps originate in Nyingchi. This result may explain the folklore of “The best Cordyceps originates in Tibet, while the best of the best Cordyceps originate in Naqu.” We also found that function prediction clustered microhabitat soil apart from the fruiting body of all the samples analyzed in the current study. This observation is compatible with previously existing hypothesis that the potential medical use of Chinese Cordyceps might be a combination of this fungus and its cohabitating microorganism. A group of pathways including D-Arginine and D-ornithnine metabolism (ko00472), fatty acid metabolism (ko00071) and glutathione metabolism (ko00480) is enriched in materials and fruiting body, between which materials seems to be more abundant of these pathways than fruiting body. However, it remains unclear if these microorganisms will have a substantial influence on consumers’ microbiome and overall health.

## Conclusions

We collected samples from five locations within the core production area of Chinese Cordyceps. Amplicon libraries of 16S rRNA genes were generated using primers targeting the V1-V3 hypervariable region and high-throughput sequencing. The results from this study suggest that for the Chinese Cordyceps-associated bacterial community there is no single core microbiome. Our data indicated that Chinese Cordyceps in the core production area of the Qinghai-Tibet Plateau have the conserved core microbiome, dominated by the phyla of both Proteobacteria and Actinobacteria. Predictive function of bacteria community was more related to the material than the host habitat location. Predictive functional profiling highlights the potential for bacterial communities in microhabitat soil to contribute chemoautotrophy and nutrient cycling, and in the external mycelial cortices involved in the biosynthesis of active constituents. This study is first used high throughput sequencing method to compare the bacterial communities inhabiting Chinese Cordyceps and its microhabitat, and to reveal composition functional capabilities of the bacteria, which will accelerate the study of the functions of bacterial communities in micro-ecological system of Chinese Cordyceps.

## Methods

### Samples collection and pretreatment

Samples collection and pretreatment was the same as described in the previous publication [9]. Chinese Cordyceps samples were collected during its early fruiting stages, in which the stroma just started to produce spores [29]. Briefly, samples of Chinese Cordyceps were collected from five locations in different dimensions including counties of Xinghai (35.85 N, 99.98 E), Qumarlêb (35.05 N, 95.14 E) and Zadoi (32.93 N, 94.98 E) in Qinghai province and counties of Biru (31.47 N, 93.63 E) and Mainling (29.21 N, 94.24 E) in Tibet Autonomous Region (Fig. 1). Five sampling locations were abbreviated as “X”, “Q”, “Z”, “Na” and “Ny” in the current study, respectively. At least 30 Chinese Cordyceps samples were collected from three random plots with 100 m apart. In each plot 5–10 samples were collected in a random manner. Collected samples were conserved in ice boxes with sterile plastic bags and transported to laboratory of Plant Biotechnology R&D Center of Shanghai Jiao Tong University quickly.

As described in our previous publication [9], all samples were divided into fruiting body (fruiting body including stromata and sclerotia, abbr. as “F”), mycoderm (microhabitat including external mycelial cortices that cover larvae, abbr. as “M”) and microhabitat soil (soil adhering to the surface of the membrane covering Chinese Cordyceps, abbr. as “S”). Abbreviated sampling location and sample names assembled the names of our sample in the current paper, for instance, sample “XF” indicated the fruiting body (abbr. as F) of Chinese Cordyceps gathered from Xinghai (abbr. as X) County. The fruiting bodies were sterilized by 75% ethanol for 2~3 min followed by 2.5% sodium hypochlorite for 20~25 min and then rinsed three times with sterile water. Samples were frozen in -20 °C until the genomic DNA was extracted for further metagenomics analyses of Chinese Cordyceps-inhabiting bacterial communities.

### Bacterial genomic DNA extraction

To ensure the homogeneity of the sequencing results, for each sample of different sampling areas, at least 30 Chinese Cordyceps samples were mixed together. To improve the genomic DNA extraction efficiency, each tissue sample was homogenized by grinding in liquid nitrogen to promote lysis of Gram-positive bacteria, thereby enhancing total DNA yields prior to the DNA extracted. Then the total genomic DNA from each sample was isolated with PowerSoil™ soil DNA Isolation Kits (Mo Bio Laboratories, USA) according to the manufacturer’s instructions. Amount and quality of isolated genomic DNA were determined with NanoDrop 2000 (Thermo Scientific, USA) and the agarose gel electrophoresis. Genomic DNA was conserved at − 80 °C prior to amplification of 16S rRNA genes.

### PCR amplification of 16S rDNA and high-throughput sequencing

For bacterial community analysis, the hypervariable V1 and V3 region of 16S rRNA gene sequence were amplified by PCR using universal primer set 8F (5′-AG-AGTTTGATCCTGGCTCAG-3′) and 536R (5′-GWATTACCGCGGCKGCTG-3′) [30]. In order to distinguish among each sample, a unique barcode containing 12 nucleotides was added in the forward primer for each sample. The PCR amplification reaction was described before [11]. Briefly, the PCR amplification was carried out in a total volume of 25 μL of PCR mixture, including 12.5 μL of Ex *Taq* DNA polymerase (Takara, Japan), 1 μL of bovine serum albumin (25 mg/mL), 1 μ mol/L (each) primer, 1 μL of template DNA, and 8.5 μL of ultrapure water. The thermal cycling program was conducted as follow: initial denaturation step was operated at 95 °C for 5 min; then 30 cycles with 30 s at 94 °C, 30 s at 52 °C, 1 min at 72 °C was conducted; finally, a 10 min elongation step at 72 °C finished the thermal program. The PCR amplifications were conducted in triple and pooled together to minimize the PCR bias. Then, the PCR products were identified by agarose gel electrophoresis and the appropriate fragments were purified with a DNA Gel Extraction Kit (Axygen, USA). After the concentration of PCR products were assayed by Qubit®2.0 Fluorometer with Qubit dsDNA HS Assay kit (Life Technologies, Invitrogen division, Darmstadt, Germany), PCR amplications were pooled equimolarly and then a pair-end library was constructed and high-throughput sequencing was performed on a Miseq PE250 platform (Illumina, USA) with MiSeq Reagent Kit v3 (Illumina) according to the instructions by Shanghai Genenergy Biotechnology Co. Ltd.

### Quantitative PCR

Quantitative PCR were carried out based on SYBR Green I. Universal primer set 338F (5′-ACTCCTACGGGAGGCAGCAG-3′) and 536R [30] were employed to quantify the copy numbers of 16S rRNA gene in each sample. The PCR reaction system and the thermal program were same with published before [10]. The data were analyzed using MxPro qPCR software version 3.0 (Stratagene, USA).

### Sequence processing and statistical analysis

Sequences data were processed using QIIME, version 1.7.0 [31] with methods similar as described before [9]. Briefly, sequences with the average quality value of each base pair lower than 20, sequence length less than 50 bp and sequences containing obscure nucleotide base in end region were removed. And then, the high-quality sequences were merged with 10 bp in the minimum overlap region and the mismatch rate was 0.2. Totally, 591,672 high-quality reads were obtained with 24,204 reads at least in each sample. Then the contig sequences were chimera-checked with USEARCH61 [32] against the database of SILVA ribosomal RNA gene database [33]. Then the quality sequences were clustered into OTUs at the similarity of 90, 95, and 97%, respectively. Representative sequences of each OTU were blasted against the Sliver database [33] to obtain the taxon of each OTU.

Alpha diversity of a bacterial community in each sample was represented by the index of Shannon-Weiner, Chao1, ACE and Simpson, which were calculated after the reads were normalized to the minimum reads (24,204 reads) in each sample. The distance between bacterial communities in any pairs of the sample was indicated with Weighted UniFrac distances [34]. Software MeV, version 4.9.0 [35] was employed to create the hierarchical clustering graph of the bacterial community in each sample by using the HCL-Hierarchical clustering method [36]. PCA was operated with the R programme. Comparison of bacterial communities was performed based on the Weighted UniFrac distance matrices by the command of “beta diversity through plots.py”. Unpaired two-tailed Student’s t-test was employed to apply statistical analysis. A *p*-value less than 0.05 was considered statistically significant.

### Prediction and analysis of gene functions of the bacterial microbiota

PICRUSt software package was used to predict predictive functional profiling of microbial communities using 16S rRNA marker gene sequences [18]. The metagenomic functions and pathways are predicted against KEGG pathways. Heatmap was generated using the online tool Morpheus at the website of https://software.broadinstitute.org/morpheus/. Hierarchical Cluster was generated based on the Spearman rank correlation. Differentially abundant functions were calculated by LDA with a *p*-value less than 0.05. The LDA score above 30 for material and 20 for different location is presented. The calculation was done by LefSe [37].

## Additional files

Additional file 1: **Figure S1.** Rarefaction curves of bacterial community inhabiting Chinese Cordyceps collected from five areas. Figure S1 (A), (B) and (C) were the OTU numbers related with the sequence number in sample of fruiting body, mycoderm and microhabitat soil, respectively. Figure S1 (D), (E) and (F) were the Shannon diversity index related with the sequence number in sample of fruiting body, mycoderm and microhabitat soil, respectively. Samples name were the same with described in Fig. 1 (TIF 413 kb)

Additional file 2: **Figure S2.** Differentially presented pathway heatmap predicted by PICRUSt. Samples name were the same with described in Fig. 1 (TIF 7807 kb)

Additional file 3: **Figure S3.** Differentially presented module heatmap predicted by PICRUSt. Samples name were the same with described in Fig. 1 (TIF 17475 kb)

Additional file 4: **Table S1.** Similarity matrix of bacterial community composition based on weighted UniFrac method. Samples name were the same with described in Fig. 1 (DOCX 18 kb)

## Abbreviations

KEGG: Kyoto encyclopedia of genes and genomes
OTUs: Operational taxonomic units
PCA: Principal component analysis
PCR: Polymerase chain reaction
PICRUSt: Phylogenetic Investigation of Communities by Reconstruction of Unobserved States
qPCR: Quantitative PCR
TEs: Transposable elements
