# Supplementary figures and images for: Composition and predictive functional analysis of bacterial communities inhabiting Chinese Cordyceps insight into conserved core microbiome
Source: BMC Microbiol. 2019 May 23;19:105. doi: 10.1186/s12866-019-1472-0 (PMC6533680; doi:10.1186/s12866-019-1472-0)

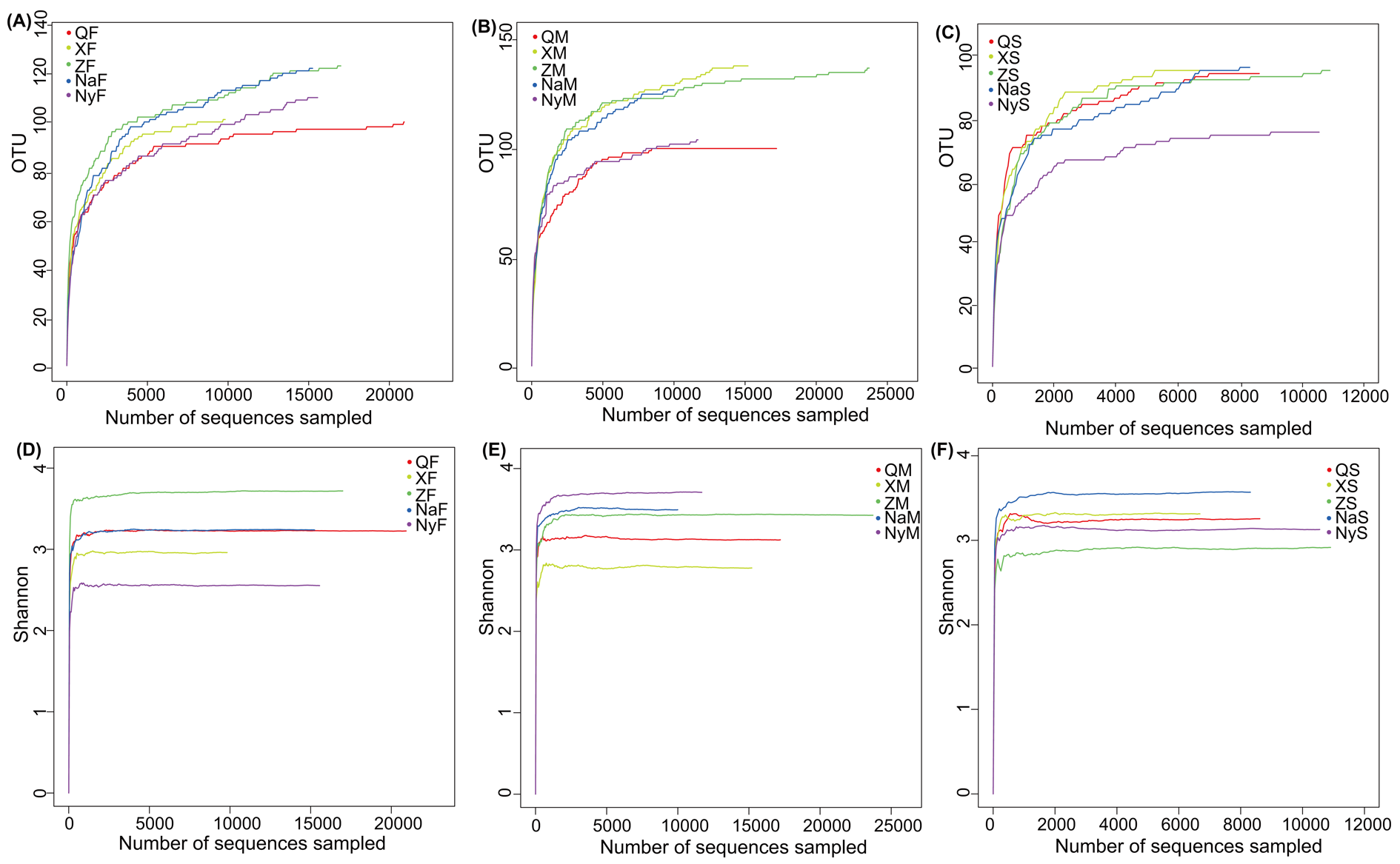

Supplement: Supplementary file 1 — Figure S1. Rarefaction curves of bacterial community inhabiting Chinese Cordyceps collected from five areas. Figure S1 (A), (B) and (C) were the OTU numbers related with the sequence number in sample of fruiting body, mycoderm and microhabitat soil, respectively. Figure S1 (D), (E) and (F) were the Shannon diversity index related with the sequence number in sample of fruiting body, mycoderm and microhabitat soil, respectively. Samples name were the same with described in Fig. 1 (TIF 413 kb) [file 12866_2019_1472_MOESM1_ESM.tif]

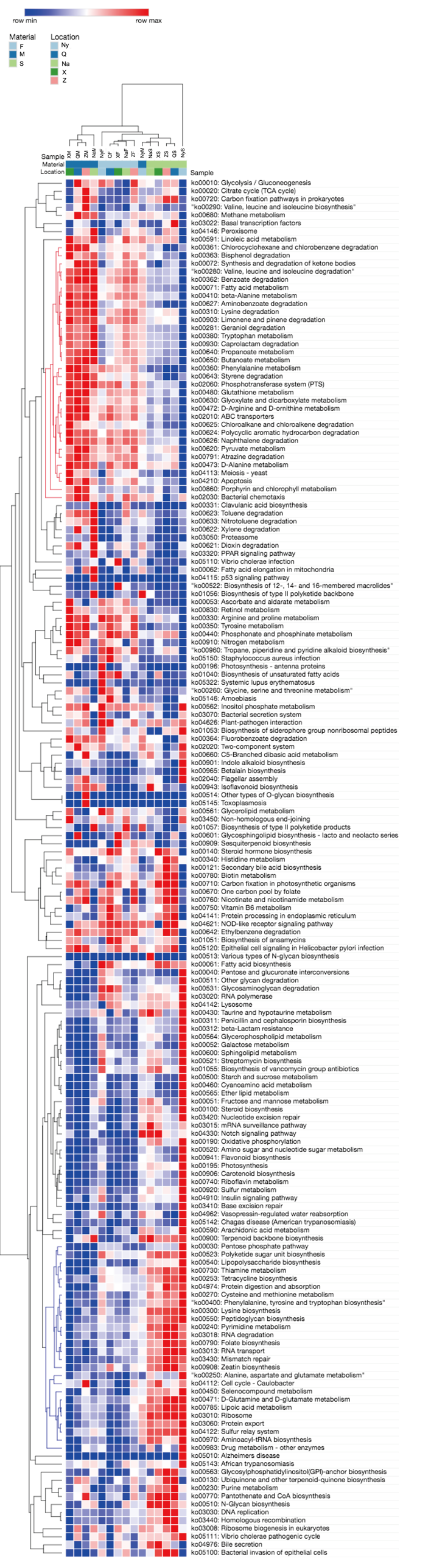

Supplement: Supplementary file 2 — Figure S2. Differentially presented pathway heatmap predicted by PICRUSt. Samples name were the same with described in Fig. 1 (TIF 7807 kb) [file 12866_2019_1472_MOESM2_ESM.tif]
