# Supplementary material for: Composition and predictive functional analysis of bacterial communities inhabiting Chinese Cordyceps insight into conserved core microbiome
Source: BMC Microbiol. 2019 May 23;19:105. doi: 10.1186/s12866-019-1472-0 (PMC6533680; doi:10.1186/s12866-019-1472-0)
Supplement: Supplementary file 4 — Table S1. Similarity matrix of bacterial community composition based on weighted UniFrac method. Samples name were the same with described in Fig. 1 (DOCX 18 kb) [file 12866_2019_1472_MOESM4_ESM.docx]

**Table S1 Similarity matrix of bacterial community in each sample of Chinese Cordyceps**

|  | QF | QM | QS | XF | XM | XS | ZF | ZM | ZS | NaF | NaM | NaS | NyF | NyM | NyS |
| --- | --- | --- | --- | --- | --- | --- | --- | --- | --- | --- | --- | --- | --- | --- | --- |
| QF | 1.0000 |  |  |  |  |  |  |  |  |  |  |  |  |  |  |
| QM | 0.4825 | 1.0000 |  |  |  |  |  |  |  |  |  |  |  |  |  |
| QS | 0.5863 | 0.5047 | 1.0000 |  |  |  |  |  |  |  |  |  |  |  |  |
| XF | 0.5789 | 0.5531 | 0.6051 | 1.0000 |  |  |  |  |  |  |  |  |  |  |  |
| XM | 0.2950 | 0.4812 | 0.2762 | 0.4061 | 1.0000 |  |  |  |  |  |  |  |  |  |  |
| XS | 0.4279 | 0.5058 | 0.5469 | 0.6069 | 0.3703 | 1.0000 |  |  |  |  |  |  |  |  |  |
| ZF | 0.5474 | 0.5852 | 0.5657 | 0.6069 | 0.4010 | 0.5029 | 1.0000 |  |  |  |  |  |  |  |  |
| ZM | 0.4009 | 0.6323 | 0.3955 | 0.4791 | 0.5230 | 0.4473 | 0.5362 | 1.0000 |  |  |  |  |  |  |  |
| ZS | 0.5867 | 0.4489 | 0.8209 | 0.5359 | 0.2390 | 0.5133 | 0.5213 | 0.3619 | 1.0000 |  |  |  |  |  |  |
| NaF | 0.5807 | 0.5166 | 0.5928 | 0.5415 | 0.3650 | 0.4728 | 0.6457 | 0.4479 | 0.5528 | 1.0000 |  |  |  |  |  |
| NaM | 0.3759 | 0.4982 | 0.4002 | 0.4444 | 0.4387 | 0.4870 | 0.4677 | 0.5099 | 0.3672 | 0.5675 | 1.0000 |  |  |  |  |
| NaS | 0.4279 | 0.4799 | 0.5324 | 0.5484 | 0.3392 | 0.6014 | 0.4755 | 0.4491 | 0.4966 | 0.5220 | 0.6454 | 1.0000 |  |  |  |
| NyF | 0.5860 | 0.5487 | 0.5590 | 0.7034 | 0.4082 | 0.5821 | 0.5842 | 0.4450 | 0.5302 | 0.5699 | 0.4936 | 0.5835 | 1.0000 |  |  |
| NyM | 0.4315 | 0.4865 | 0.4712 | 0.5149 | 0.3494 | 0.5670 | 0.4995 | 0.4351 | 0.4252 | 0.5088 | 0.5817 | 0.6286 | 0.6026 | 1.0000 |  |
| NyS | 0.3880 | 0.3782 | 0.5371 | 0.4890 | 0.2764 | 0.5199 | 0.3961 | 0.3458 | 0.5202 | 0.4283 | 0.4875 | 0.6630 | 0.5434 | 0.5631 | 1.0000 |
